# Exposure to a slightly sweet lipid-based nutrient supplement during early life does not increase the level of sweet taste most preferred among 4- to 6-year-old Ghanaian children: follow-up of a randomized controlled trial

**DOI:** 10.1093/ajcn/nqy352

**Published:** 2019-03-27

**Authors:** Harriet Okronipa, Mary Arimond, Charles D Arnold, Rebecca R Young, Seth Adu-Afarwuah, Solace M Tamakloe, Maku E Ocansey, Sika M Kumordzie, Brietta M Oaks, Julie A Mennella, Kathryn G Dewey

**Affiliations:** 1 Program in International and Community Nutrition, Department of Nutrition, University of California, Davis, CA; 2 *Intake* - Center for Dietary Assessment, Washington, DC; 3 Department of Nutrition and Food Science, University of Ghana, Legon, Ghana; 4 Department of Nutrition and Food Sciences, University of Rhode Island, Kingston, RI; 5 Monell Chemical Senses Center, Philadelphia, PA

**Keywords:** lipid-based nutrient supplement, sweet taste preference, Monell forced-choice test, children, Ghana

## Abstract

**Background:**

The impact of feeding a slightly sweet nutrient supplement early in life on later sweet taste preference is unknown.

**Objective:**

We tested the hypothesis that the level of sucrose most preferred by 4–6-y-old children exposed to a slightly sweet lipid-based nutrient supplement (LNS) early in life would not be higher than that of children never exposed to LNS.

**Design:**

We followed up children born to women (*n* = 1,320) who participated in a randomized trial in Ghana. In one group, LNS was provided to women on a daily basis during pregnancy and the first 6 mo postpartum and to their infants from age 6 to 18 mo (LNS group). The control groups received daily iron and folic acid or multiple micronutrients during pregnancy and the first 6 mo postpartum, with no infant supplementation (non-LNS group). At age 4–6 y, we randomly selected a subsample of children (*n* = 775) to assess the concentration of sucrose most preferred using the Monell 2-series, forced-choice, paired-comparison tracking procedure. We compared LNS with non-LNS group differences using a noninferiority margin of 5% weight/volume (wt/vol).

**Results:**

Of the 624 children tested, most (61%) provided reliable responses. Among all children, the mean ± SD sucrose solution most preferred (% wt/vol) was 14.6 ± 8.6 (LNS group 14.9 ± 8.7; non-LNS group 14.2 ± 8.4). However, among children with reliable responses, it was 17.0 ± 10.2 (LNS group 17.5 ± 10.4; non-LNS group 16.5 ± 10.0). The upper level of the 95% CI of the difference between groups did not exceed the noninferiority margin in either the full sample or those with reliable responses, indicating that the LNS group did not have a higher sweet preference than the non-LNS group.

**Conclusion:**

Exposure to a slightly sweet nutrient supplement early in life did not increase the level of sweet taste most preferred during childhood. This trial was registered at clinicaltrials.gov as NCT00970866.

## Introduction

Evidence suggests that all children are born with the ability to detect sweet tastes (1, 2), and that children prefer higher levels of sweetness than adults (3). The degree to which early exposure modulates sweetness preferences later in life remains largely unknown. The few studies that have examined the long-term impact of early exposure to sweetness on later sweet taste preferences have been observational (4–6). Compared with little or no history of feeding sugar water, the feeding of water or teas sweetened with table sugar, Karo syrup, or honey during early infancy has been associated with a greater preference for sweetened water at age 6 mo (4) and 2 y (5), and for more concentrated sucrose solutions at age 6–10 y (6). However, the continued feeding of other sweet foods and beverages beyond early infancy may confound these observed associations.

Various types of supplements have been evaluated as part of strategies to address undernutrition during the first 1,000 days of life. One category of such supplements is small-quantity lipid-based nutrient supplements (LNS), which are made from vegetable oil, milk powder, peanut paste, sugar, and multiple micronutrients (MMN), and have been shown to have the potential to improve child growth and development in some contexts (7–11). They have a slightly “sweet” taste due to the added sugar (e.g., ∼1.6 g/20 g LNS) and milk sugar they contain, and acceptance and reported adherence have been good (11–15). There is no evidence that this small amount of sugar consumption by the mother would alter the sweetness of amniotic fluid or breastmilk, and it is also unlikely that it would affect the taste preferences of the offspring when given directly to the child. Nonetheless, given that LNS are novel products, any potential consequences with regard to sweet taste preferences need to be investigated. To our knowledge, no studies have examined the long-term impact of providing sweet-tasting supplements early in life, during sensitive periods of flavor learning (16), on sweet taste preference during later childhood. Examining this question in lower-middle-income countries is important because many of them are undergoing a nutritional transition involving the increased intake of foods/beverages high in added sugar and a rise in obesity rates, including among children (17).

To evaluate the long-term impact of early exposure to LNS, we conducted a follow-up study with a cohort of children who had participated in the International Lipid-based Nutrient Supplement (iLiNS) DYAD trial in Ghana between 2009 and 2014. During the trial LNS was provided to women during pregnancy and the first 6 mo postpartum and to their infants from age 6 to 18 mo in one of the intervention groups. We used a psychophysical tool validated for inclusion in the NIH Toolbox (18, 19) that allowed us to directly measure sweet taste among children and not rely solely on maternal reports. We first determined the ability of 4–6-y-old children to comprehend the task in the field setting. We then tested the hypothesis that the level of sweetness most preferred among children who were exposed to LNS early in life would not be higher than that of children who were never exposed to LNS using a noninferiority approach to rule out potential adverse effects, in accordance with the principle of “first do no harm”.

## Methods

### Design of the parent trial

The iLiNS DYAD-Ghana trial was a community-based, partially double-blind, individually randomized controlled trial conducted between 2009 and 2014 in 2 semi-urban districts (Yilo and Manya Krobo) in the Eastern region of Ghana, located about 70 km north of the capital, Accra. The trial was designed to examine the efficacy of a small quantity of LNS (20 g) for the prevention of malnutrition in pregnant and lactating women and their infants. Details of the study design, randomization, and recruitment have been reported elsewhere (12). Briefly, pregnant women at ≤20 weeks of gestation were randomly assigned to 1 of 3 groups. One group (LNS group) received 20 g of LNS daily during pregnancy and the first 6 mo postpartum; women typically ate the palatable, slightly sweet supplement (containing 22 micronutrients and 118 kcal) mixed with any food of their choice. From 6 to 18 mo of age, mothers were instructed to feed their infants 20 g LNS daily either mixed with other foods or alone (11). Each 20 g of LNS supplement contained 4 g of total sugars with 1.2 g and 1.6 g of added sugar in the maternal and child versions, respectively. The children in the other 2 groups are combined herein (non-LNS group) because neither they nor their mothers received any food supplements. Their mothers ingested nonflavored capsules containing either iron and folic acid during pregnancy and a low dose of calcium for 6 mo postpartum, or MMN during pregnancy and the first 6 mo postpartum. Although all mothers received basic nutritional advice, none of the mothers in either group received instructions to limit the added sugar intake of their infants. As reported previously, LNS was added to the food of the children in the LNS group an average of 73.5% of the days between 6 and 18 mo of age (11).

Sociodemographic information at the time of enrollment into the parent trial was collected by means of a questionnaire and included details of: maternal age, education, marital status and nulliparity, household food insecurity, and household assets. We constructed a household assets score based on ownership of a set of assets (radio, television, refrigerator, cell phone, and stove), lighting source, drinking water supply, sanitation facilities, and flooring materials. The household asset score was created using principal components analysis and had a mean of 0 and SD of 1, with higher values representing a higher socioeconomic status. We assessed the feeding practices of infants and young children, including caregiver reports of the child's consumption of food and beverage items, at multiple time points between birth and 18 mo. This was achieved through a guided free recall of liquids and foods consumed by the child on the day before the interview and a list‐based recall of the number of days that each food group was consumed in the 7 d preceding the interview (20). The questionnaire had a total of 32 food and beverage items; each item was part of a list of items belonging to a food group or food category (e.g., fruits, dark green vegetables, sugary foods). There were 6 sugary food and beverage items in total (i.e., fruit juice or any juice drinks; chocolate or cocoa drink without milk; chocolate or cocoa drink with milk; yogurt; soft drinks or any other sweet drink; sugary foods such as chocolates, sweets, candies, pastries, cakes, or sweet biscuits). In this article, we report the proportion of children who consumed a sugary food or a sugary beverage the day preceding the day of interview when the child was 9 and 18 mo of age.

### Recruitment into the follow-up study

**FIGURE 1:**
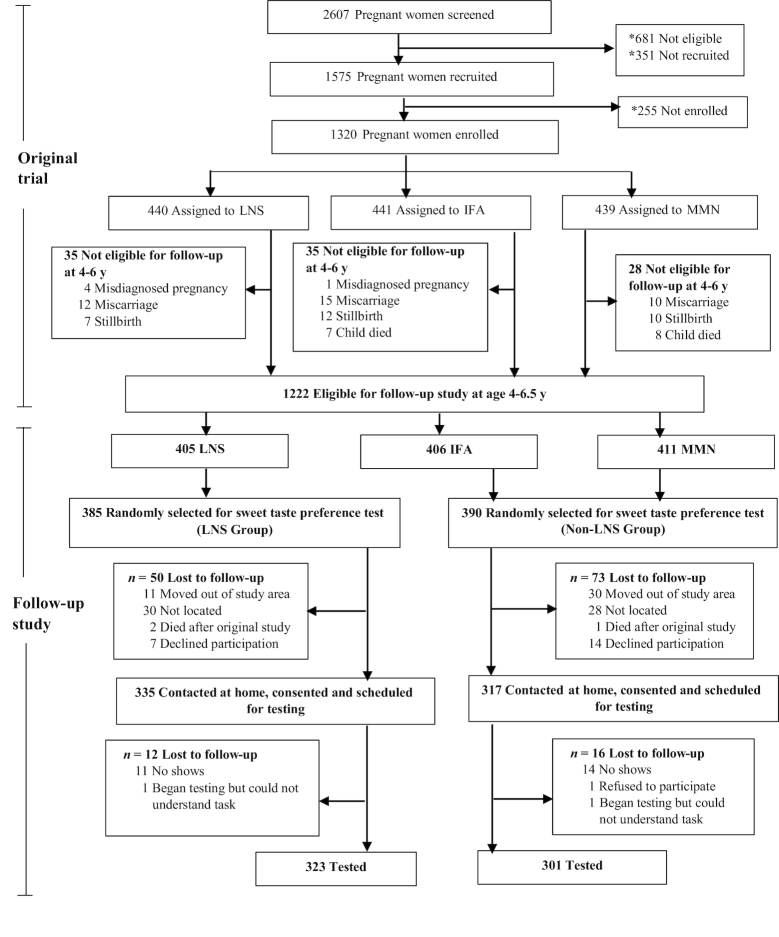
Study profile. LNS group, women received 20 g LNS daily during pregnancy and 6 mo lactation. Infants received 20 g LNS daily from 6–18 mo of age; non-LNS group, women received either IFA during pregnancy and placebo for 6 mo postpartum or multiple micronutrient (MMN) capsules during pregnancy and 6 mo lactation. Infants did not receive any supplement. LNS, lipid-based nutrient supplement; IFA, iron and folic acid. *Details reported in (12).

When children were 4-6 y of age, we conducted a follow-up (January to December 2016) of the participants of the iLiNS DYAD-Ghana trial to determine the long-term impact of early nutritional supplementation on health and nutrition outcomes, which included sweet taste preferences, food and beverage preferences, and consumption among the children. All children whose mothers participated in the parent trial were eligible for the follow-up study regardless of whether or not they or their mothers were lost to follow-up before the end of the parent trial. Excluding child deaths, miscarriages, and stillbirths, 1,222 children were eligible to participate in the follow-up study (**Figure 1**). The present study on sweet taste preference includes a randomly selected subsample of children (*n* = 775) selected from the 1,222 who were eligible.

We contacted mothers or caregivers (in cases where the child was in the care of someone other than the mother) mostly by phone to inform them of the follow-up study. If they were interested, study personnel went to the home to provide more details of the study procedures and to obtain informed consent. If consent was obtained, the test visit was scheduled. We reimbursed each mother (or caregiver) for transportation costs on the day of testing. Mothers and study personnel were not informed of the study hypothesis and study personnel were blind to group assignment of the children. All study protocols were approved by the Institutional Review Board of the University of California, Davis, the Ethics Committee for the College of Basic and Applied Sciences at the University of Ghana, and the Ghana Health Service Ethical Review Committee. The follow-up study was part of the iLiNS DYAD-Ghana trial, which was registered at clinicaltrials.gov as NTC00970866.

### Psychophysical testing procedures

We used the Monell 2-series, forced-choice, paired-comparison tracking procedure to determine the concentration of sucrose most preferred. This tool is ideal for testing in pediatric populations because it requires a short time to complete, does not require verbal communication of responses, and controls for position bias (19, 21, 22).

Five concentrations of sucrose solution were prepared [3%, 6%, 12%, 24%, 36% weight/volume (wt/vol), which is equivalent to 0.09, 0.18, 0.35, 0.70, 1.05 M]. These sucrose solutions were prepared every 1–2 d and stored refrigerated; the refrigerated solutions were brought to room temperature prior to testing. During taste testing, the sucrose solutions were presented to the child in small disposable medicine cups; distilled water was used for rinsing the mouth between pairs and between series and a bucket was provided for spitting; a stopwatch was used to monitor inter-pair and inter-series intervals; and data were recorded on a tracking grid, as described in Mennella et al. (19, 22). The test instructions on the grid were translated into 3 common local languages (i.e., Krobo, Ewe, and Twi).

Testing took place in a closed room at our testing center which was easily accessible by public transportation. The room was ventilated and eating was not allowed in the room to minimize food odors. The room was partitioned into sections, one of which was used to familiarize the children with the testing procedures in the presence of the mother/caregiver. The mother/caregiver remained silent in this room during the testing and was out of view of the child (to prevent any distraction). Testing of children occurred in the other sections of the room, each of which was staffed by a trained research assistant. The child sat on a chair behind a small table designed for children. One research assistant conducted the study and the other monitored the timing of inter-pair and inter-series intervals using a stopwatch and, based on the child's response, selected the pairs of sucrose solutions for testing (see below). As a check for following correct procedures, each assistant monitored the child's responses and both had to agree on the next pair of samples given to the child or when the child reached criterion.

Details of the test have been described elsewhere (19, 21). In brief, following fasting for at least 1 h, participants were presented with pairs of solutions (5 mL each) in medicine cups that differed in sucrose concentration. The first pair presented was from the middle range of concentrations (6% and 24% wt/vol). The child tasted each solution within a pair for 5 s without swallowing and then pointed to the solution they preferred, without instruction on how the stimuli differed. They rinsed their mouths once between each sample and twice between each pair during an enforced 1-min interval. Each subsequent pair of solutions presented contained the concentration selected by the child in the preceding pair and an adjacent concentration stimulus. This pattern continued until the child chose the same concentration when paired with both a higher and lower concentration in two consecutive pairs or chose the highest or lowest concentration twice consecutively.

After a 3-min break, we repeated the entire task but stimulus pairs were presented in the reverse order (in the protocol, for series 1 the lower concentration was presented first; for series 2 the higher concentration was presented first). This controls for position bias and enables researchers to determine objectively whether the child understands the task or is responding by pointing to whatever is presented to their right or left (19, 21). The geometric mean of the concentrations selected in series 1 and 2 provides the estimate of the most preferred concentration of sucrose.

### Child anthropometry

Height was measured in duplicate to the nearest 0.1 cm using a stadiometer (Seca 217; Seca) and weight was measured in duplicate to the nearest 50 g using a Seca scale (Seca 875; Seca). Using the WHO Anthro software (23), we calculated BMI-for-age z-scores of the children.

### Sample size calculation and statistical analysis

Originally, our sample size was calculated based on detecting a small effect size of 0.2 (24) between groups in the concentration of sucrose most preferred and in other outcomes in the follow-up study (to be reported elsewhere). This yielded a minimum sample size of 775 (388 per group) assuming an α = 0.05 and 80% power, an SD of 8.2% (obtained from a pilot study to test the feasibility of the tool to examine sweet taste preference in 30 children who did not participate in the parent trial, conducted prior to the start of the follow-up study), and up to 25% attrition.

We posted a statistical analysis plan on the project website (www.ilins.org) prior to data analysis. We used a noninferiority approach to compare the sweet taste preference between the intervention and control groups. The primary outcome was the concentration of sucrose most preferred. We chose 5% wt/vol as our noninferiority margin based on one study that reported a mean difference of 6% wt/vol in the concentration of sucrose most preferred at age 6–10 y between children who were routinely fed sugar water during infancy when compared with similarly aged children who were rarely or minimally exposed to sugar water during infancy (6). A 5% wt/vol difference in sucrose solution between groups translates to 5 g sugar in 100 mL water. To detect this mean difference of 5% between groups, we needed a minimum sample size of 336 (168 per group) (α = 0.05, power = 0.9), calculated based on published data (19) from which we determined the most preferred concentration of sucrose (17.1% ± 11.0% wt/vol; mean ± SD) among only those participants who were aged between 5 and 7 y (J Mennella, Monell Chemical Senses Center, Philadelphia. Personal communication, 2017). The actual target sample size of 775 allowed for the possibility that some children might not understand the sweet taste test instructions and their data might have to be excluded in sensitivity analyses.

We first determined how many children understood the task by categorizing children based on the reliability of their responses between series 1 and 2. Responses were considered reliable if the choice made in series 2 was the same as or ≤2 steps away from the choice made in series 1, and unreliable if the choice made in series 2 was ≥3 steps away from the choice made in series 1. Second, we determined whether the geometric mean of the two series differed between the groups by conducting 2 separate analyses. The first analysis included all children and the second included only children who exhibited reliable responses.

We examined differences between treatment groups using both negative binomial and linear regression modeling techniques. Since results from the analytical methods were similar, we present the results from the linear regression models for ease of interpretation. Noninferiority was deemed to be established if the 95% CI of the difference between the treatment groups fell below the noninferiority margin.

ANOVA or chi-squared tests were used to determine whether the groups differed in maternal, child, or household characteristics. Potential prespecified covariates were considered for covariate adjustment if they were significantly associated with the outcome (*P* < 0.10). These included child sex as well as maternal characteristics assessed at baseline (prior to enrollment into the parent trial) (12): years of formal education, marital status, age, estimated prepregnancy BMI, and nulliparity; a household assets index derived from a principal components analysis (25); household food insecurity access scale (26); distance in meters to the nearest weekly market; and main language spoken at home. All models included child age at the time of testing. We examined the potential interaction between child age and intervention group with regard to level of sweetness most preferred. Data analysts were fully blinded to group assignments until analyses were completed. All analyses were conducted using SAS for Windows Version 9.4 (SAS Institute).

## Results

### Participants

The study profile is shown in Figure 1. Of the 775 children randomly selected for participation, 15.9% (*n* = 123) could not be re-enrolled mostly because they could not be contacted. Of the 652 who were re-enrolled and scheduled for testing, 624 were tested: 301 from the LNS group and 323 from the non-LNS group. Most child and household characteristics did not differ between children who participated in the study and those who were lost to follow-up (**Supplemental Table 1**). However, mothers of children who were lost to follow-up were younger, less likely to be married, more likely to be nulliparous prior to the parent trial, and had lower prepregnancy BMI than those who were tested at follow-up.

At the time of testing, children ranged in age from 4.0 to 6.5 y (4–4.9 y, *n* = 320; 5–6.5 y, *n* = 304). As shown in **Table 1**, the groups did not differ in maternal and household baseline characteristics nor in child characteristics at follow-up, except for the percentage of households in which Krobo was the main language spoken. The consumption of sugary foods and beverages was common at 9 and 18 mo of age, and did not differ by intervention group (Table 1).

**FIGURE 2:**
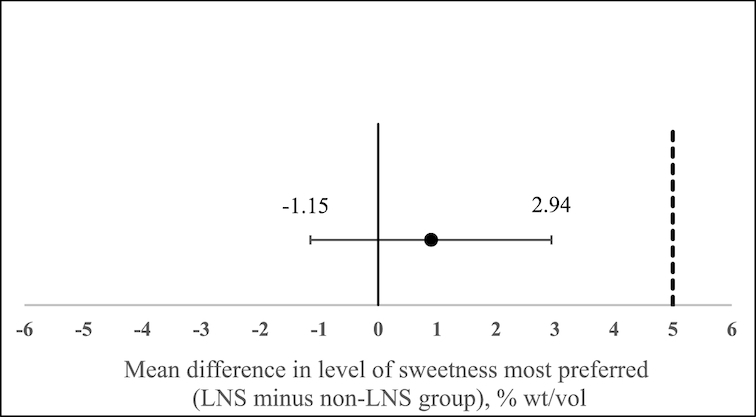
Difference in sucrose concentration most preferred between the LNS and non-LNS groups (for children with reliable responses). Error bars indicate 95% CIs. The noninferiority margin is denoted by the dotted line. The 95% CIs lie to the left of the noninferiority margin (5% wt/vol), indicating noninferiority (that is, the concentration of sucrose most preferred by the LNS group was not higher than that preferred by the non-LNS group). LNS, lipid-based nutrient supplement; non-LNS, no exposure to LNS (control group).

**TABLE 1:** Maternal and child characteristics by intervention group for children who participated in the iLiNS DYAD-Ghana follow-up study and had sweet taste data^1^

Variable^2^	All groups combined (*n* = 624)	LNS group (*n* = 323)	Non-LNS group (*n* = 301)	*P* value^3^
Maternal characteristics at time of enrollment into the parent trial				
Age, y	26.9 ± 5.6	26.9 ± 5.6	26.8 ± 5.5	0.786
Education, y	7.7 ± 3.6	7.6 ± 3.7	7.9 ± 3.5	0.315
Married or cohabiting *n* (%)	579 (92.8)	299 (92.6)	280 (93.0)	0.827
Prepregnancy BMI, kg/m^2^	24.7 ± 4.4	24.9 ± 4.5	24.5 ± 4.3	0.170
Nulliparity *n* (%)	204 (32.7)	104 (32.2)	100 (33.2)	0.785
Household speaks Krobo as main language *n* (%)	455 (72.9)	248 (76.8)	207 (68.8)	0.024
Household Assets Score^4^	0.0 ± 0.9	−0.1 ± 0.9	0.1 ± 0.9	0.066
Household Food Insecurity Access Scale^5^	2.5 ± 4.0	2.2 ± 3.8	2.8 ± 4.2	0.089
Distance to market, m	1965 ± 1902	2020 ± 1941	1907 ± 1862	0.456
Children's exposure to sweets, ages 9 and 18 mo				
Consumption of sugary food at age 9 mo^6^*n* (%)	151 (25.9)	79 (26.5)	72 (25.3)	0.731
Consumption of sugary beverage at age 9 mo^7^*n* (%)	130 (22.2)	61 (20.3)	69 (24.2)	0.259
Consumption of sugary food at age 18 mo^6^*n* (%)	310 (51.8)	151 (49.2)	159 (54.6)	0.182
Consumption of sugary beverage at age 18 mo^7^*n* (%)	324 (54.2)	167 (54.4)	157 (53.9)	0.913
Child characteristics at follow-up				
Sex *n* (%) male	304 (48.72)	155 (48.0)	149 (49.5)	0.705
Age, y	5.0 ± 0.6	5.1 ± 0.6	5.0 ± 0.6	0.385
Height, cm	106.8 ± 5.5	106.8 ± 5.7	106.7 ± 5.2	0.960
Weight, kg	16.6 ± 2.2	16.7 ± 2.3	16.6 ± 2.1	0.435
BMI-for-age z-score, BMIZ	−0.57 ± 0.82	−0.55 ± 0.81	−0.60 ± 0.82	0.491

^1^LNS, lipid-based nutrient supplement; non-LNS, no exposure to LNS (control group).

^2^Values are means ± SDs or*n* (%).

^3^Group differences were compared using ANOVA for continuous variables and the chi-squared test for categorical variables.

^4^Proxy indicator for household socioeconomic status; higher values represent higher socioeconomic status.

^5^Proxy indicator for household food insecurity; higher values represent higher food insecurity.

^6^Defined as child having consumed a sweetened food item the day preceding the interview, as reported by caregiver.

^7^Defined as child having consumed a sweetened beverage item the day preceding the interview, as reported by caregiver.

### Task performance

Of the 627 who arrived at the facility for testing, 1 child refused to participate and 2 children did not comprehend the instructions (Figure 1). As shown in **Table 2**, 624 of the 627 children completed both series 1 and 2. The majority of the children (61%) chose either the same concentration of sucrose or the concentrations differed by ≤2 steps in series 1 compared with 2. However, 39% (*n* = 243) of the children responded unreliably (39.0% in the LNS group compared with 38.9% in the non-LNS group, *P* = 0.972). That is, the solution most preferred in the second series differed by ≥3 steps from the one most preferred in the first series, which could be evidence of a random choice or position bias (e.g., the child picked which came first in both series). The children who gave unreliable responses were younger than the others (4.8 ± 0.5 compared with 5.1 ± 0.6 y, *P* < 0.001). Half (52%, 167/320) of the 320 children younger than 5 y, and more than two-thirds of the children older than 5 y (70.4%, 214/304) understood the task and gave reliable responses.

**TABLE 2:** Task performance by intervention group, reflecting completion and comprehension of the psychophysical taste task among children who participated in the iLiNS DYAD-Ghana follow-up study^1^

	All groups combined *n* = 624	LNS group *n* = 323	Non-LNS group *n* = 301
Number of pairs (trials) required to reach criterion	7.5 ± 1.4	7.5 ± 1.5	7.5 ± 1.4
Agreement between series 1 and 2, *n* (%)			
Chose the same solution in series 1 and 2	116 (18.6)	60 (18.6)	56 (18.6)
1 step apart	179 (28.7)	93 (28.8)	86 (28.6)
2 steps apart	86 (13.8)	44 (13.6)	42 (13.9)
>2 steps apart (not reliable; random choice)	243 (38.9)	126 (39.0)	117 (38.9)

^1^LNS, lipid-based nutrient supplement; non-LNS, no exposure to LNS (control group). Values are means ± SDs or *n* (%).

There were no differences between the LNS and non-LNS groups in the number of sucrose concentration pairs needed to reach criterion (*P* = 0.974) or the length of the test session (*P* = 0.389). On average, children required 7.5 ± 1.5 presentations to reach criterion and the test duration was 14.4 ± 2.8 min.

### Most preferred concentration of sucrose by intervention group

For all children (*n* = 624) who completed testing, the most preferred sucrose concentration was 14.6% ± 8.6% wt/vol (**Table 3**, LNS, 14.9% ± 8.7% wt/vol; non-LNS, 14.2% ± 8.4% wt/vol). Of those children who gave reliable responses (*n* = 381), the most preferred sucrose concentration was 17.0% ± 10.2% wt/vol (Table 3, LNS, 17.5% ± 10.4% wt/vol; non-LNS, 16.5% ± 10.0% wt/vol). The upper end of the CI for the difference in means between groups for either the full sample (+1.95% wt/vol, Table 3) or the sample of children who gave reliable responses (+2.94% wt/vol, Table 3, **Figure 2**) did not cross our noninferiority margin of 5% wt/vol, suggestive of a noninferiority finding, i.e., children in the LNS group did not have a higher sweet taste preference than children in the non-LNS group.

**TABLE 3:** Mean sucrose concentration most preferred, by intervention group among children who participated in the iLiNS DYAD-Ghana follow-up study^1^

	LNS group	Non-LNS group	LNS vs. no LNS difference in means (95% CI)^2^	*P* value^3^
All children				
*n*	323	301	—	
Sucrose concentration most preferred, % wt/vol	14.9 ± 8.7	14.2 ± 8.4	0.61 (−0.73, 1.95)	0.372
Sucrose concentration most preferred, molarity	0.43 ± 0.25	0.41 ± 0.24	0.02 (−0.02, 0.06)	
Children with reliable responses^4^				
*n*	197	184	—	
Sucrose concentration most preferred, % wt/vol	17.5 ± 10.4	16.5 ± 10.0	0.90 (−1.15, 2.94)	0.389
Sucrose concentration most preferred, molarity	0.51 ± 0.30	0.48 ± 0.29	0.03 (−0.03, 0.08)	

^1^iLiNS, International Lipid-based Nutrition Supplement; LNS, lipid-based nutrient supplement; non-LNS, no exposure to LNS (control group). Values are means ± SDs.

^2^Differences between groups were tested using multiple linear regression.

^3^Models were adjusted for child age at testing. No other variable was included in the models apart from child age.

^4^Children whose choice for series 1 was the same or was 1 or 2 concentrations away from their choice for series 2.

Maternal, household, and child characteristics examined were not associated with the sucrose concentration most preferred in bivariate analysis and so these characteristics were not adjusted for in models comparing the groups. We found no significant interaction between child age and intervention group with regard to the most preferred sucrose concentration in the analysis including the full sample (*P* = 0.509) and the subset of children who provided reliable responses (*P* = 0.733).

## Discussion

In this follow-up of the iLiNS DYAD trial cohort in Ghana, we observed that the daily provision of a slightly sweet food supplement (LNS) to mothers during pregnancy and 6 mo postpartum, and to their infants from age 6–18 mo did not impact sweet taste preference at age 4–6 y: the sucrose concentration most preferred by children in the LNS group was not higher than that most preferred by the children who received no LNS supplement. To the best of our knowledge, this is the first study to examine the long-term impact of early and prolonged exposure to LNS on sweet taste preference of children later in life.

On average, 4–6-y-old Ghanaian children most preferred a 17% wt/vol sucrose solution. To put this in perspective, this is equivalent to approximately 10 teaspoons of sugar in 237 mL of water (i.e., an 8-oz glass), nearly twice the sugar concentration of a typical cola. Our results are consistent with studies of racially diverse children living in the United States that used the same psychophysical method (19, 22). The average sucrose concentration most preferred was 18% wt/vol in one study among 5–9.9-y-old children (19), and 20% wt/vol in another study among 5–10-y-old children (22).

Decades of basic research have shown that children are born with the ability to detect and prefer sweet tastes (3), presumably to attract them to the predominant taste quality of the milk of their mother and then to sources of energy (carbohydrates) during a period of rapid growth (27, 28). When compared with adults, children most prefer higher levels of a variety of sugars, both nutritive (19, 29) and nonnutritive (30), with the adult-like preference pattern emerging during mid-adolescence (19, 31). For this and other reasons, we did not expect sweet taste preference to shift due to the provision of a slightly sweet supplement. Moreover, the LNS we used in our study contributed only a small percentage of the overall sugar intake; children were instructed to consume 20 g of LNS per day which contained 4 g of total sugar including 1.6 g of added sugar. A high proportion of children in both groups were already consuming sugary foods and beverages as early as ages 9 and 18 mo, which is consistent with data from the 2008 Ghana Demographic and Health Survey showing that >30% of children aged 6–23 mo were already consuming sugary foods the day or night prior to the interview (32). Thus, apart from the LNS we provided, exposure to other sugary foods and beverages at an early age was high and did not differ between the groups, which could explain why we found no group differences in the concentration of sweet taste most preferred.

Our finding of no difference in the sucrose concentration most preferred between children who were exposed to the slightly sweet supplement and those who were not may appear to be contrary to findings from observational studies in the United States (4–6). These studies suggested that children routinely fed sugar water or sweetened teas during infancy preferred a more concentrated sugar solution at 6 mo (4), 2 y (5), and 6–10 y (6) of age compared with children with no history of being fed sugar water. For example, the latter study, which used the same psychophysical method to measure sweet preference as in the present study, showed that children who were routinely fed sugar water and sweetened teas as infants mostly preferred a 23% wt/vol sucrose solution at 6–10 y, significantly higher than those with little or no such feeding history (16% wt/vol). The level of sucrose most preferred in our Ghanaian cohort was much lower than the level most preferred by children routinely fed sugar water in the above study.

There are several potential explanations for the differences in results between our study and these other studies. First is the difference in study designs. The above studies were observational in nature and thus confounding factors may explain the findings. As argued by Mennella and Bobowski (33), such associations between feeding sugar water during infancy and heightened sweet preferences during childhood may reflect feeding practices related to sweetened foods and beverages that persist as the child grows and not be the result of early taste programming per se. No compelling data exist to suggest that repeated exposure to sweetened water results in a generalized heightened hedonic response to sweetness in foods and beverages (5, 34). Our study was a randomized controlled trial which means that potential factors that could influence the outcome were likely to be balanced between intervention groups.

Second, the type of exposure or the context in which sweetness was experienced differed between studies. Whereas the main exposure in the observational studies was sweetened water, the exposure in our study was a slightly sweet food supplement. It is possible that experience with a sweetened food such as LNS may not generalize to presentations of solutions of sucrose in water. Apart from the fact that all children biologically tend to prefer sweet foods, they also learn what should or should not taste sweet through familiarization (34, 35), which can be influenced by various factors including the dietary patterns and cultural practices of the family (36–43). Repeated exposure to specific flavors or specific taste in food influences the preference for that food or specific taste as well as similarly flavored foods (44–46). For example, children who were assigned to taste sweetened tofu (an unfamiliar food) repeatedly over several weeks preferred that version over salted and plain versions in a postexposure taste test (35).

Third, there may have been differences between studies in the sweetness level of the exposure. Although information on quantities of sugar fed and the intensity of sweetness was not reported in the observational studies, it is likely that the level of sweetness as well as the amount of added sugar consumed in the sweetened water or teas was higher than that consumed from the LNS in our study. Fourth, the studies differed with regard to timing of exposure. In our study, mothers were instructed to feed LNS directly to their children starting from age 6 mo, whereas in the above-mentioned studies, mothers generally started feeding sweetened water to their children much earlier, before age 6 mo. Thus, exposure to sweetened water could have occurred during early sensitive periods of flavor learning, which may have had a greater impact on later preferences for sugar water.

Our study had a number of strengths and weaknesses that deserve mention. We followed the same cohort that had participated in the trial from early pregnancy to age 18 mo, then at age 4–6 y. We were able to re-establish contact with a majority of eligible study participants for follow-up at age 4–6 y. As a result, our study had a large sample size and was therefore appropriately powered to test a noninferiority hypothesis. The field personnel who conducted the test and the primary analyst were blinded to the intervention group of study participants. The 2 intervention groups remained balanced across most maternal, child, and household characteristics. Additionally, we used an objective, validated, and reliable method to directly measure sweet taste preference in children (19). The 2-series test we used had an in-built check for positional bias which made it possible to objectively identify children with inconsistent responses. About one-third of our sample (mostly younger children) gave inconsistent responses in their sucrose concentration most preferred for series 1 and 2, an indication that they most likely did not understand the test instructions. However, their exclusion from the analyses did not change our findings. We also observed that compared with children who were tested at follow-up, mothers of children who were lost to follow-up were younger, had lower prepregnancy BMI, were less likely to be married, and more likely to be nulliparous. However, none of these factors were associated with the most preferred sucrose concentration and adjusting for them (data not shown) did not change our conclusions, so our findings should be generalizable to our study population. In addition, the questionnaire we used to collect information on sugary food and drink consumption when the children were 9 and 18 mo of age was not validated in our study population. However, the questionnaire was an adapted version of the infant and young child feeding practices questionnaire developed by WHO for global use. As recommended in WHO 2010 (47), the questionnaire was adapted for local use based on our knowledge of the diets and food culture of the study area. Lastly, we did not conduct any sensory tests to objectively determine the perceived sweetness of LNS, which limits the extent to which we can compare these results to those of previous studies.

We conclude that exposure to a slightly sweet nutrient supplement, LNS, early in life did not increase sweet taste preference later in childhood in this semi-urban setting in Ghana. We recommend that further studies examine this question in populations with a lower level of exposure to other sweet foods and drinks during infancy and preschool years, and also evaluate whether long-term exposure to LNS impacts children's preference for and consumption of specific foods and beverages later in life.

## Supplementary Material

nqy352_Supplemental_File

## Notes

Supplemental Table 1 is available from the “Supplementary data” link in the online posting of the article and from the same link in the online table of contents at https://academic.oup.com/ajcn/.

Abbreviations used: iLiNS, International Lipid-based Nutrient Supplement; MMN, multiple micronutrients; LNS, lipid-based nutrient supplement; wt/vol, weight/volume.
