# Supplementary material for: Exposure to a slightly sweet lipid-based nutrient supplement during early life does not increase the level of sweet taste most preferred among 4- to 6-year-old Ghanaian children: follow-up of a randomized controlled trial
Source: Am J Clin Nutr. 2019 Mar 27;109(4):1224–32. doi: 10.1093/ajcn/nqy352 (PMC6462430; doi:10.1093/ajcn/nqy352)
Supplement: nqy352_Supplemental_File [file nqy352_supplemental_file.docx]

Exposure to a slightly sweet lipid-based nutrient supplement during early life does not increase the level of sweet taste most preferred among 4 to 6-year-old Ghanaian children: follow-up of a randomized controlled trial

| **Supplemental Table 1: Maternal and child characteristics at baseline and follow-up for children who participated in the iLiNS-DYAD Ghana follow-up study and were included in the analysis compared to those who were lost to follow-up** | | | |
| --- | --- | --- | --- |
| **Variable^1^** | **Tested at follow-up**  **N=624** | **Lost to follow-up**  **N=151** | **P-value^2^** |
| Mothers characteristics at time of enrolment into the parent trial |  |  |  |
| Age (y) | 26.9 ± 5.6 | 25.2 ± 4.9 | 0.001 |
| Education (y) | 7.7 ± 3.6 | 7.9 ± 3.8 | 0.498 |
| Married or cohabiting % (n/N) | 92.7 (579/624) | 85.4 (129/151) | 0.004 |
| Maternal pre-pregnancy BMI (kg/m^2^) | 24.7 ± 4.4 | 23.7 ± 3.9 | 0.015 |
| Nulliparity % (n/N) | 32.7 (204/624) | 45.7 (69/151) | 0.003 |
| Household speaks Krobo as main language % (n/N) | 72.9 (455/624) | 68.2 (103/151) | 0.248 |
| Household Assets Score^3^ | 0.01 ± 0.97 | -0.05 ± 1.04 | 0.541 |
| Household Food Insecurity Access  Scale^4^ (#) | 2.50 ± 4.00 | 2.9 ± 4.38 | 0.296 |
| Distance to market (m) | 1965 ± 1902 | 1534 ± 1462 | 0.013 |
|  |  |  |  |
| Child characteristics |  |  |  |
| Sex n (% male) % (n/N) | 48.7 (304/624) | 50.0 (65/130) | 0.790 |
| ^1^Data are presented as mean ± SD or n/N (%); n, number of participants identified as “yes” for the variable in question; N, total number of participants in the group in question.  ^2^Group differences were compared using ANOVA for continuous variables and the chi-squared test for categorical variables.  ^3^Proxy indicator for household socioeconomic status; higher values represent higher socioeconomic status.  ^5^Proxy indicator for household food insecurity; higher values represent higher food insecurity. | | | |
